# Application of Flexible Micro Temperature Sensor in Oxidative Steam Reforming by a Methanol Micro Reformer

**DOI:** 10.3390/s110202246

**Published:** 2011-02-16

**Authors:** Chi-Yuan Lee, Shuo-Jen Lee, Chia-Chieh Shen, Chuin-Tih Yeh, Chi-Chung Chang, Yi-Man Lo

**Affiliations:** 1 Department of Mechanical Engineering, Yuan Ze Fuel Cell Center, Yuan Ze University, Taoyuan 320, Taiwan; E-Mails: mesjl@saturn.yzu.edu.tw (S.-J.L.); ccshen@saturn.yzu.edu.tw (C.-C.S.); s975055@mail.yzu.edu.tw (C.-C.C.); s995048@mail.yzu.edu.tw (Y.-M.L.); 2 Department of Chemical Engineering and Materials Science, Yuan Ze Fuel Cell Center, Yuan Ze University, Taoyuan 320, Taiwan; E-Mail: ctyeh@saturn.yzu.edu.tw

**Keywords:** micro reformer, micro temperature sensor, OSRM

## Abstract

Advances in fuel cell applications reflect the ability of reformers to produce hydrogen. This work presents a flexible micro temperature sensor that is fabricated based on micro-electro-mechanical systems (MEMS) technology and integrated into a flat micro methanol reformer to observe the conditions inside that reformer. The micro temperature sensor has higher accuracy and sensitivity than a conventionally adopted thermocouple. Despite various micro temperature sensor applications, integrated micro reformers are still relatively new. This work proposes a novel method for integrating micro methanol reformers and micro temperature sensors, subsequently increasing the methanol conversion rate and the hydrogen production rate by varying the fuel supply rate and the water/methanol ratio. Importantly, the proposed micro temperature sensor adequately controls the interior temperature during oxidative steam reforming of methanol (OSRM), with the relevant parameters optimized as well.

## 1. Introduction

Methanol is a more feasible liquid fuel for use in a reformer owing to its high safety of storage and transport, high carbon/hydrogen ratio, high power density and low reforming temperature (250–300 °C) [1]. However, the technology application-related problems related to its use include starting time and power density. Also, how to produce hydrogen efficiently and steadily is another important concern.

The ability of the proposed micro sensor to monitor the inner environment of a micro methanol reformer requires the following features: a high sensitivity, short response and recovery time, reproducibility and long stability, low interference (high selectivity), simplified process and mass production capability, wide monitor range, low cost, and low energy waste.

Liu and Yang have used polyimide as the substrate material for a flow sensor [2,3], while Chuang fabricated a micro temperature sensor and micro heater on poly-dimethylsiloxane (PDMS) film [4]. Bielska [5] used polyamide foil as the basic material and fabricated gold and copper electrode on it by using micro-electro-mechanical systems (MEMS) technology. The temperature is evaluated at a range of 30 °C to 42 °C, and the resistance change is used based on the temperature to study its linear outcome.

Zhang [6] fabricated platinum/titanium on Pyrex 7740 glass based on the use of MEMS technology, while simultaneously using the heater as temperature sensor. Platinum and titanium are then deposited using an E-beam evaporator, followed by completion of the sensor shape by using the lift-off method.

Based on use of MEMS technology, Shih [7] fabricated platinum/titanium on a silicon wafer to be used in temperature gradient interaction chromatography, in which a layer of parylene is deposited around the sensor to become an air gap to increase thermal isolation and decrease heat loss.

Yan [8] used nickel-chromium alloy as the material of a micro heater and nickel as the material for a temperature sensor. An electron probe micro analyzer was also used to evaluate the adhesion between nickel and the substrate material when depositing nickel on different substrate materials. According to their results, depositing nickel on silicon nitride and silicon oxide is better than depositing nickel on silicon wafers.

Xiao [9] developed an inexpensive and flexible micro temperature sensor by using low pressure chemical vapor deposition to deposit phosphosilicate glass to be the sacrificial layer. A layer of polyimide was also coated, followed by use of MEMS technology to fabricate platinum on polyimide. The subsequently flexible sensor can remove the sacrificial layer and does not limit the position of a sensor.

Despite macromolecular advantages such as thermal stability, high dimensional stability, and low permittivity, the temperature change is slightly too large. This work attempts either to prevent the film and basic material from flaking off or to decrease the yield rate from the heat stress by using stainless steel foil for a flexible base material of 40 μm thickness. Stainless steel foil must have high corrosion resistance, high compression resistance, high temperature resistance and high flexibility. This work attempts to decrease the interference of a methanol reformer and diagnose the inner environment immediately by using thin metal foil as the substrate material of the proposed micro sensor.

## 2. Methodology

This section comprises descriptions of a micro flow plate and a micro temperature sensor.

### 2.1. Design of a Micro Flow Plate

The micro flow plate is used in various technologies, in which the appearance of a flow changes with the miniaturization of a flow plate. Thorough elucidation of the characteristics of a flow would increase the scope of potential applications. Design of a flow channel inside the micro reformer must consider the following factors: heat conduction rate and methanol/air mixing rate, pressure decrease and pressure distribution, catalyst reaction area and chemistry reaction flow direction, flow rate distribution, mass transfer rate and concentration distribution, as well as viscous energy energy-dissipating and divergence heat transfer [10]. Based on the above factors, the design of a planner flow channel is adopted, as described in the following section

### 2.2. Design of a Micro Temperature Sensor

Conventionally adopted sensors can be classified as contactless or contactable, depending on their sensing mode. Contactable sensors can be classified as resistance temperature detectors, thermistors, thermocouples and mercury thermometers. Additionally, contactless sensors can be categorized into pyroelectric and quantum temperature sensors. Regardless of whether a contactable or contacless sensor is considered, too large of a volume interferes with the flow and increases the complexity of packaging, making it impossible for them to integrate with a micro channel. Given that a small volume, high precision, production batch and low cost characterize micro sensors based on MEMS technology, this work presents a flexible micro temperature sensors based on this technology.

**Figure 1.**
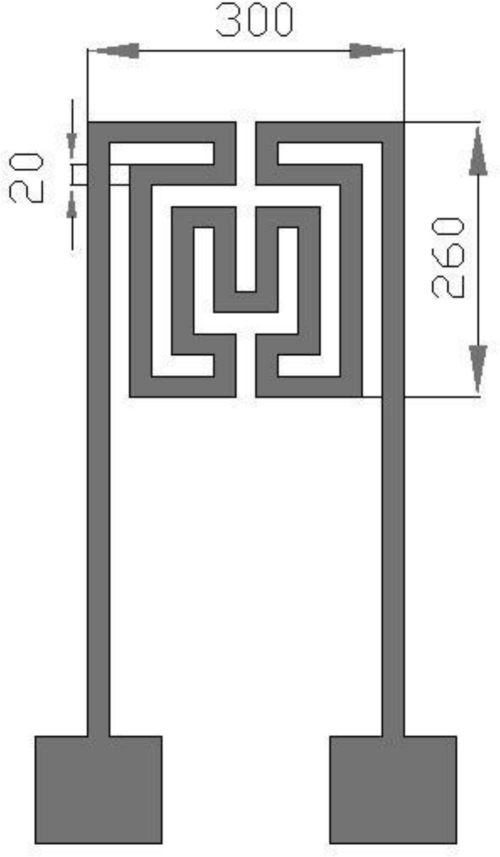
Electrode dimensions of a micro temperature sensor (units: μm).

**Figure 2.**
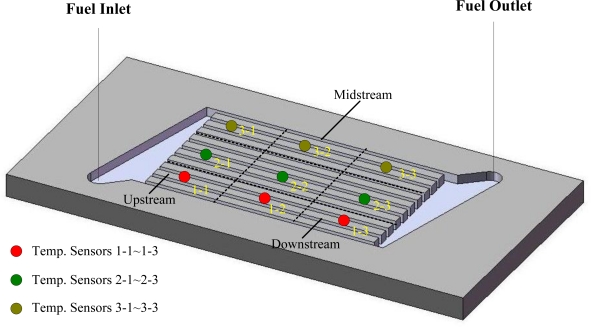
Micro temperature sensor distribution.

This work develops a flexible micro temperature sensors based on MEMS technology to measure inner temperature distributions. The measurement area is increased by placing micro sensors in a reaction area intensively. Additionally, the size of a micro temperature sensor is decreased to measure the temperature variation of a small area and decrease the thickness of sensing membranes in order to increase the reaction rate. Electrode length of the proposed micro temperature sensor is 260 μm and the width is 300 μm; both the line width and interval are 20 μm as shown in Figure 1. Three micro temperature sensors are placed in one group, in which nine groups are set at the upstream, midstream and downstream of the micro flow plate, as well as one group at the inlet and one at the outlet. Figure 2 shows the micro temperature sensors distribution.

### 2.3. Fabrication of a Micro Flow Plate

**Figure 3.**
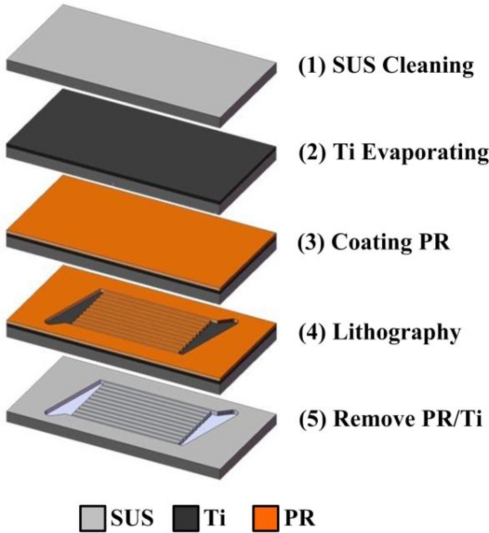
Process sketch of a micro flow plate.

**Figure 4.**
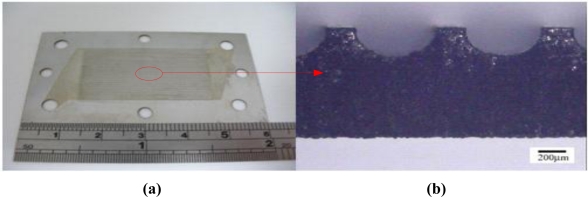
Planer micro flow plate: **(a)** surface; **(b)** sectional.

A micro flow plate is manufactured based on MEMS technology, in which SUS 304 is used as the substrate and the thickness of SUS 304 is 600 μm. Figure 3 illustrates the process. An E-beam evaporator is used to deposit 500 Å of titanium on stainless steel to function as a sticking layer, followed by use of spin coater coating Hexamethyldisilazane (HMDS) as the sticking layer for the photoresist. Photoresist AZ-4620 is then coated on stainless steel to function as the etching mask. Next, an aligner and mask are used to make a transition graph for the photoresist, followed by use of MP2500 + H_2_O to define the pattern. Following development, titanium and aqua regia are etched using hydrofluoric acid to etch stainless steel, followed by removal of the titanium mask and photoresist. Next, pattern is cut to the desired shape by wire cutting. Figure 4 illustrates the finished product.

### 2.4. Fabrication of a Flexible Micro Temperature Sensor

The material of the micro temperature sensor is gold, and 40 μm thick stainless steel foil was used as the substrate. Regardless of whether wet etching or metal lift-off is used, a layer of insulating material is coated, explaining our use of RF sputter to grow 1,000 Å of aluminum nitride (AlN).

**Figure 5.**
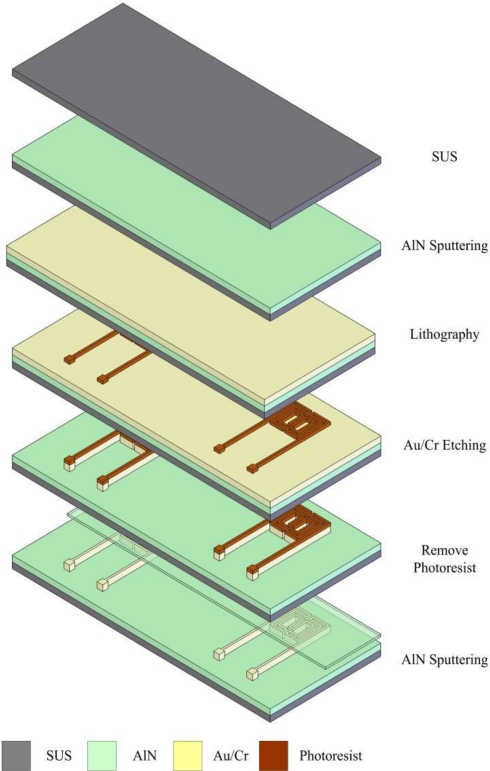
Process sketch of a micro temperature sensor.

**Figure 6.**
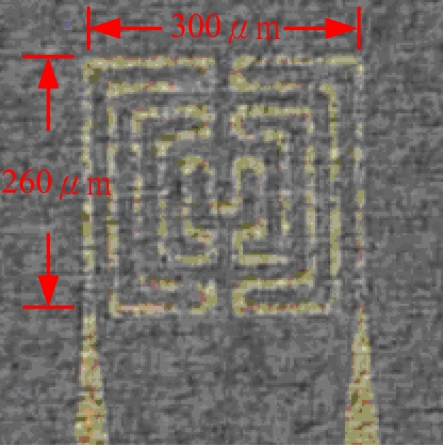
Optical microscope picture of a micro temperature sensor.

**Figure 7.**
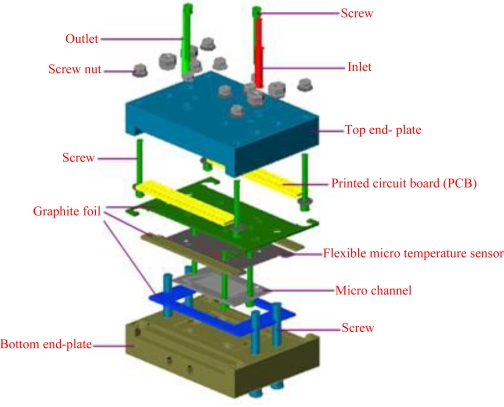
Structure of the micro reformer combined with flexible micro temperature sensors.

Next an E-beam evaporator is used to deposit 200 Å of chromium to be sticking layer. 2,000 Å of gold is then deposited. Next, photolithography is performed to define the pattern of a micro temperature sensor, followed by use of etching liquid to etch gold. Finally, fabrication of the micro temperature sensor is completed after removing the photoresist layer. Figure 5 shows the micro temperature sensor, while Figure 6 shows the optical microscope diagram of the micro temperature sensor. Figure 7 schematically depicts the structure of the micro reformer combined with flexible micro temperature sensors.

## 3. Results and Discussion

### 3.1. Inner Temperature Distribution of a Micro Temperature Sensor during OSRM Reaction at 270 °C

**Figure 8.**
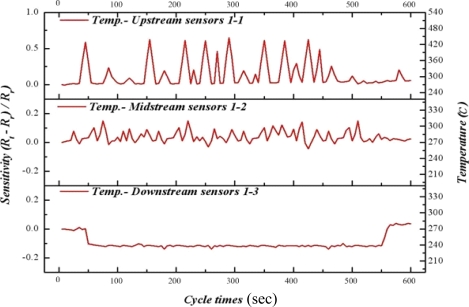
Inner temperature distribution of micro reformer at area 1-1, 1-2, 1-3 (OSRM; 270 °C).

**Figure 9.**
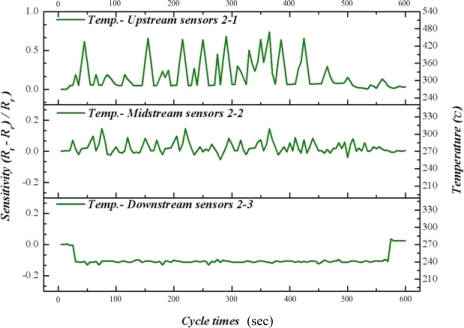
Inner temperature distribution of micro reformer at area 2-1, 2-2, 2-3 (OSRM; 270 °C).

**Figure 10.**
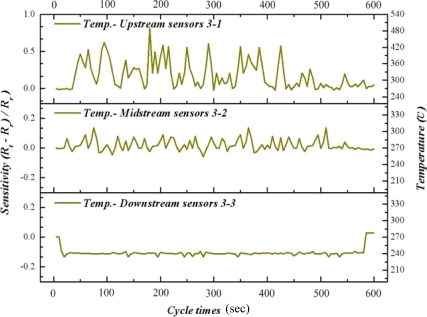
Inner temperature distribution of micro reformer at area 3-1, 3-2, 3-3 (OSRM; 270 °C).

In this study, CuPd/CeZn was selected as a reforming catalyst. The composition of catalyst is CuO-35wt%, PdO-2wt%, CeO_2_-20wt%, and ZnO_2_-43wt%. The catalyst (30 mg) was washcoated on a micro channel [11]. Figures 8 to 10 illustrate the upstream, midstream and the downstream temperatures of inserted flexible micro temperature sensors during an oxidative steam reforming of methanol (OSRM) reaction. Under the same operating environment, methanol conversion can achieve 100% at 270 °C, as long as the temperature is maintained during inner measurements. The upstream reveals that the temperature responsively rolls acutely, normally ending in 1–3 cycles. We posit that the inner reaction transforms into an exothermic reaction in a relatively short time.

Although the maximum temperature of temperature responsively is approximately 550 °C, it does not appear often; in addition, the second high temperature ranges from 270 °C to 450 °C. The midstream temperature responsively is about 0, indicating that it is during the reciprocation between endothermic reaction and exothermic reaction. The temperature distribution is at 260 °C–300 °C. The downstream shows endothermic reaction directly, in which the temperature variation is around 250 °C–260 °C.

From Figures 8 to 10, we can infer that fuel entering the micro channel burns with the oxygen at upstream, in which a large number of exothermic reactions lead to partial oxidation reaction of methanol. When it reaches midstream, the consumption of oxygen makes it come before the oxidative steam reforming reaction of methanol, revealing a balance between endothermic reaction and exothermic reaction. Then, at downstream, a catalyst reaction changes to the steam reforming reaction of methanol completely. The exothermic reaction of upstream and midstream offer some heat to the downstream, and the fuel consumption does not reveal an obvious endothermic reaction.

### 3.2. Inner Temperature Distribution of a Micro Temperature Sensor during OSRM Reaction at 300 °C

**Figure 11.**
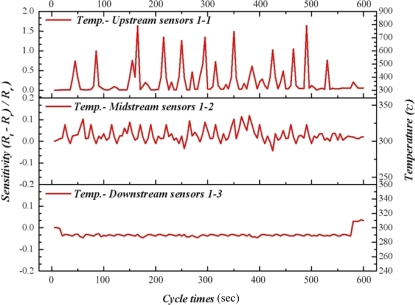
Inner temperature distribution of micro reformer at area 1-1, 1-2, 1-3 (OSRM; 300 °C).

**Figure 12.**
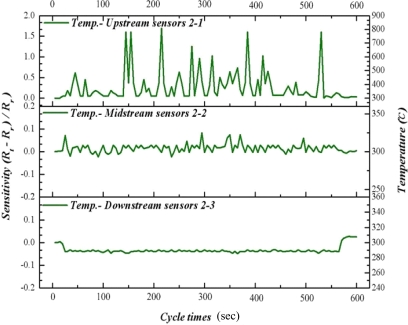
Inner temperature distribution of micro reformer at area 2-1, 2-2, 2-3 (OSRM; 300 °C).

**Figure 13.**
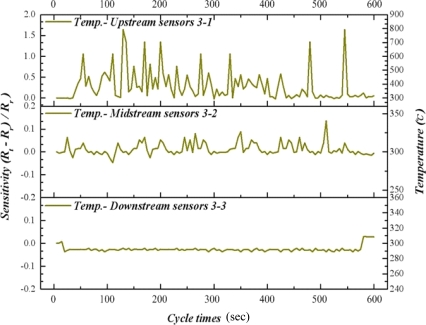
Inner temperature distribution of micro reformer at area 3-1, 3-2, 3-3 (OSRM; 300 °C).

As time advances, our results indicate that the methanol conversion rate has decreased gradually, implying decreasing catalyst activity. Therefore, raising the reaction temperature to 300 °C facilitates the catalyst reactivation. Figures 11 to 13 reveal that the upstream, midstream and the downstream processing undergo partial oxidation of methanol (POM) and oxidative steam reforming of methanol (OSRM) and steam reforming of methanol (SRM) obviously. The maximum temperature upstream is approximately 800 °C, in which the average temperature is around 450 °C. However, the reaction time is extremely short.

## 4. Conclusions

This work successfully integrates a micro reformer and flexible micro temperature sensors to alleviate the pressurization problem. Heat tolerance of the printed circuit board is also increased, which involves the bonding breakout of printed circuit boards in order to devise a standardized procedure. Micro temperature sensors contribute significantly to the partial measurement in a micro methanol reformer by measuring more deeply than an external measurement does. This work successfully integrates micro temperature sensors inside the micro methanol reformer to measure the inner condition the reformer immediately, ultimately providing further insight into the inner temperature distribution during the oxidative steam reforming reaction of methanol.
